# Molecular Identification and Characterization of Five *Ganoderma* Species from the Lower Volta River Basin of Ghana Based on Nuclear Ribosomal DNA (nrDNA) Sequences

**DOI:** 10.3390/jof10010006

**Published:** 2023-12-21

**Authors:** Gideon Adotey, Raphael N. Alolga, Abraham Quarcoo, Paul Yerenkyi, Phyllis Otu, Abraham K. Anang, Laud K. N. Okine, Winfred S. K. Gbewonyo, John C. Holliday, Vincent C. Lombardi

**Affiliations:** 1 Science Laboratory Department, Accra Technical University, Barnes Road, P.O. Box GP 561, Accra 00233, Ghana; qabraham@atu.edu.gh (A.Q.); paulwin98@yahoo.com (P.Y.); phyllisotu@gmail.com (P.O.); 2 State Key Laboratory of Natural Medicines, Department of Pharmacognosy, China Pharmaceutical University, Nanjing 210009, China; alolgara@cpu.edu.cn; 3 Noguchi Memorial Institute for Medical Research (NMIMR), University of Ghana, Accra 00233, Ghana; aanang@noguchi.ug.edu.gh; 4 Department of Biochemistry, Cell and Molecular Biology (BCMB), University of Ghana, Accra 00233, Ghana; lkokine@yahoo.com (L.K.N.O.); wsgbew@ug.edu.gh (W.S.K.G.); 5 Aloha Medicinals Inc., Carson City, NV 89706, USA; john@alohamedicinals.com; 6 Department of Microbiology and Immunology, School of Medicine, University of Nevada, 1664 N Virginia St. MS 0320, Reno, NV 89557, USA

**Keywords:** Ganodermataceae, lucidum, reishi, molecular phylogenetic, internal transcribed spacer, nuclear large subunit

## Abstract

*Ganoderma* is a genus of biomedical fungus that is used in the development of numerous health products throughout the world. The Lower Volta River Basin of Ghana is an undulating land surface covered by extensive vegetation and water bodies and is rich in polypore mushrooms resembling various members of the *Ganoderma* genus. Despite the extensive biopharmaceutical benefits of *Ganoderma* spp., the isolates from the Lower Volta River Basin have not been properly characterized, thus limiting their use in the development of biotechnological products. In this study, *Ganoderma* spp. collected from the Lower Volta River Basin were genetically analyzed using the nuclear ribosomal sequences, the internal transcribed spacer 2 (ITS 2), the complete internal transcribed spacer (ITS), and the nuclear large subunit (nLSU). Blastn search and sequence analysis revealed that the sample we coded as Ganoderma LVRB-2 belongs to *G. mbrekobenum*, whereas Ganoderma LVRB-1, Ganoderma LVRB-14, and Ganoderma LVRB-16 belong to the species *G. enigmaticum*. Our analysis further demonstrates that Ganoderma LVRB-17 belongs to the species *G. resinaceum*. Thus, the five samples collected in the present study were positioned in three different distinct groups, namely *G. mbrekobenum*, *G. enigmaticum,* and *G. resinaceum*. The current data may serve as reference points for future studies.

## 1. Introduction

Members of the genus *Ganoderma* are economically and ecologically important white-rot fungi, with an extensive and impressive range of applications. The worldwide usage of *Ganoderma* spp. for biomedical and alternative health purposes is significant, and an ever-increasing number of products that incorporate ganoderma as an active ingredient are commercially available. They include extracts and isolated constituents in various formulations, which are marketed all over the world in the form of capsules, creams, hair tonics, and syrups [1]. The genus *Ganoderma*, comprising more than 250 species [2,3], has gained acceptance as a biomedical material for the development of many types of health products in China, Japan, and the USA [4]. In China, for example, extracts from various *Ganoderma* species have been used for adjuvant anticancer clinical therapy [5]. Several natural bioactive compounds, including polysaccharides, triterpenoids, nucleosides, sterols, alkaloids, polypeptides, fatty acids, and steroids, have been isolated from the mycelia, fruiting body, and spores of ganoderma. These compounds have been reported to demonstrate numerous biologically relevant properties, but perhaps the most significant are their immunomodulatory and antitumor activities [6,7,8].

Given the growing importance of ganoderma as a health-promoting biomedical fungus, the species used in formulating various health products must be properly identified. Until recently, the identification of different *Ganoderma* spp. was based mainly on morphological and cultural characteristics, but this method is known to be affected by pleomorphic and environmentally influenced characteristics, imposing serious limitations on their identification [9]. Furthermore, the identification of *Ganoderma* spp. based on their morphological and cultural characteristics is an experience-based science; however, most mycologists lack the necessary expertise to accurately differentiate the various species. The genetic approach, involving DNA sequence analysis, is one of the most reliable methods for the identification of *Ganoderma* spp. [10,11]; it is convenient, rapid, and accurate, and it only requires a small quantity of sample [12].

Recent biogeographic studies suggest that Africa harbors the highest diversity of *Ganoderma* spp. [13]. Unfortunately, many species have not been properly characterized to establish their phylogenetic positions in relation to *Ganoderma* spp. from other parts of the world. In Ghana, their identification is mainly performed by physical examination of the phenotypic characteristics of the fruiting bodies using the naked eye. As a result, most identified *Ganoderma* spp. from Ghana lack the supporting molecular data [14], thus making their molecular phylogenetic positions dubious. Recently, the Food Research Institute of Ghana, in collaboration with the University of Minnesota, conducted molecular phylogenetic studies on *Ganoderma* spp. collected from the Brong Ahafo and Upper West regions of Ghana. This collaborative research led to the identification and naming of three new *Ganoderma* spp.: *G. wiiroense* [15], *G. mbrekobenum*, and *G. enigmaticum* [16]. Our goal, as presented in this communication, was to characterize the various ganoderma samples collected from the Lower Volta River Basin (LVRB) of Ghana through Bayesian phylogenetic analysis, a statistical inference approach for estimating species phylogeny and divergence.

## 2. Materials and Methods

### 2.1. Chemicals and Reagents

Antibiotic malt extract agar (AMEA) and malt extract agar (MEA) were from Fungi Perfecti, LLC (Olympia, WA, USA); UltraClean Plant DNA Isolation Kits were from MoBio Laboratories, Inc. (Carlsbad, CA, USA); GeneClean Spin kits were from Qbiogene, Inc. (Santa Ana, CA, USA); BigDye Terminator sequencing enzyme v.3.1 was from Applied Biosystems (Foster City, CA, USA); and the PCR master mix was from Promega Corp. (Madison, WI, USA).

### 2.2. Origin and Sampling of Isolates of Ganoderma Species

**Figure 1:**
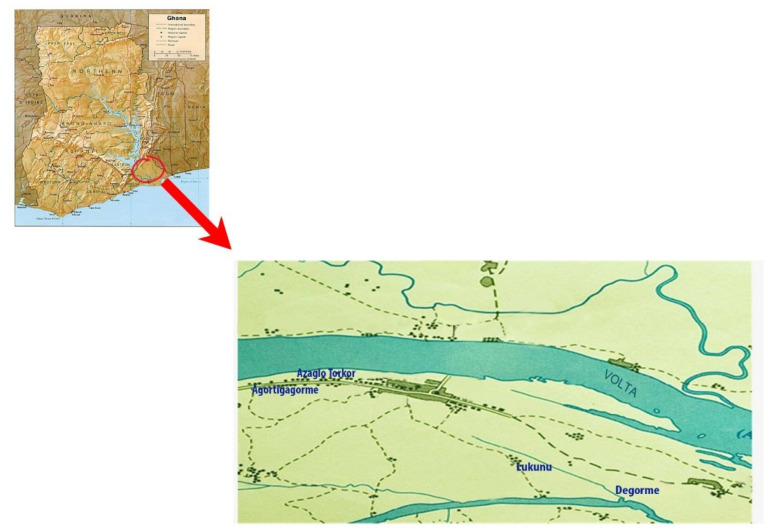
Locational map of sample collection sites in the Lower Volta River Basin of Ghana Subsection.

Fresh mushroom fruiting bodies resembling *Ganoderma* spp. were collected during mycological surveys from May to June 2015. Samples were collected from different locations (Agortigagorme, Azaglo Torkor, Lukunu, and Degorme) in Mepe and the surrounding communities, all in the Lower Volta River Basin of Ghana (Figure 1). The fresh mushroom fruiting bodies were cleaned and aseptically transferred in paper bags for transport to the Science Laboratory Technology Department of Accra Technical University for hyphae pseudoparenchyma fragment isolation.

### 2.3. Hyphae Pseudoparenchyma Fragment Isolation

The fruiting bodies of the respective isolates (Ganoderma LVRB-1, Ganoderma LVRB-2, Ganoderma LVRB-14, Ganoderma LVRB-16, and Ganoderma LVRB-17) were surface sterilized with 70% alcohol, cut longitudinally with a sterilized scalpel, and then a small piece of this sample was taken aseptically from the inner core of each fruiting body. The isolated samples were placed on AMEA in a Petri dish and incubated in the dark at 28 °C for 10 days. The resulting clean and genetically pure mycelium was transferred to MEA, consisting of 2% *w*/*v* malt extract and 1.5% *w*/*v* agar without antibiotics, and cultured for another 10 days to obtain pure mycelium from the fungal culture.

### 2.4. DNA Extraction

Genomic DNA extraction was performed following a slightly modified protocol outlined in Aime and Phillips-Mora [17]. In brief, approximately 2–4 mm^3^ of mycelium was aseptically excised from the actively growing edge of the colony and extracted using the UltraClean Plant DNA Isolation Kit (UltraClean Plant DNA Isolation Kits were from MoBio Laboratories, Inc. (Carlsbad, CA, USA) per the manufacturer’s instructions. DNA extractions with evidence of co-extracted fungal pigments were cleaned with the GeneClean Spin kit according to the manufacturer’s protocol.

### 2.5. Polymerase Chain Reactions (PCR)

The ITS2 region was amplified with the primer pairs ITS3 and ITS4 as previously described and with a slightly modified protocol [18]. Briefly, PCR was performed in 25 µL reaction mixtures, containing 12.5 µL of 2× PCR buffer, 1 µL of each primer (2.5 µM), and 2 µL of DNA extract, and the total volume was adjusted to 25 µL with sterile, deionized water. PCR amplification was conducted using the following procedure: 94 °C for 5 min, 40 cycles of 94 °C for 30 s, 50 °C for 30 s, 72 °C for 1 min, and a final extension at 72 °C for 10 min [19].

The primers ITS1F [20] and ITS4 [18] were used to amplify the internal transcribed spacer (ITS) region. The PCR amplification of the ITS ribosomal region was performed following the protocol outlined in [17], with a slight modification. Briefly, 12.5 µL of PCR master mix, 1.25 µL each of 10 mM primers (upstream and downstream), and 10 µL of diluted (10- to 100-fold) DNA template were mixed, yielding a 25 µL reaction volume [15]. Amplification was achieved with a 2 min denaturation step at 94 °C, then 35 cycles of 94 °C for 39 s, 50 °C for 15 s, and a final extension of 45 s at 60 °C [17].

The nLSU ribosomal region was amplified using the primer pairs LROR and LR6 as previously described [21], and the reaction was performed in 25 µL volumes, as described above for the ITS. LSU amplification was carried out under the same cycling program for the ITS, except the cyclic extension step was increased to 4 min. All the PCR products were cleaned using Montage^TM^ PCR Centrifugal Filter Devices, from Millipore Corp., Burlington, MA, USA [17].

### 2.6. Cycle Sequencing

Sequencing reactions were cleaned using ethanol precipitation and sequenced with BigDye Terminator sequencing enzyme v.3.1 following a modified protocol described in [17]. The sequencing was performed as follows: 2 µL of diluted BigDye in 1:3 dilutions of BigDye dilution buffer (400 mM Tris pH 8.0, 10 mM MgCl_2_); 0.3 µL of 10 mM primer; 10–20 ng of cleaned PCR template; and H_2_O to 5 µL total reaction volume. For ITS2 and ITS, the sequencing primers were the same as those used for PCR, but for LSU, LR0R, LR3R, LR5, and LR16, they were used as internal sequencing primers [22].

### 2.7. DNA Sequence Comparisons and Data Sets

The identification of the isolates based on DNA sequence data was conducted by a similarity search of the nuclear ribosomal ITS2, ITS, and LSU sequences using NCBI BLASTn (release 2.12.0) search against *Ganoderma* spp. in GenBank of the National Center for Biotechnology Information (NCBI). DNA sequences sharing at least 99% nucleotide identity with those from the Lower Volta River Basin were downloaded and used for comparison.

### 2.8. Phylogenetic Analyses

Phylogenetic trees were generated based on Bayesian inference (BI). The sequence data set generated was aligned with Clustal Omega in Geneious Prime Version 2022.01 and refinements to the alignment were executed manually. BI analysis was conducted for the ITS2, ITS, and nLSU using Mr. Bayes in Geneious Prime Version 2022.01 with the GTR model and chain length set to 1,100,000 generations.

## 3. Results

### 3.1. Origin and Sampling of Ganoderma Species

Five isolates designated Ganoderma LVRB-1, Ganoderma LVRB-2, Ganoderma LVRB-14, Ganoderma LVRB-16, and Ganoderma LVRB-17 were opportunistically collected from different locations (Agortigagorme, Azaglo Torkor, Kizito Campus, Lukunu, and Degorme) in the Lower Volta River Basin of Ghana (Figure 1) during the rainy season of May–June 2015.

**Figure 2:**
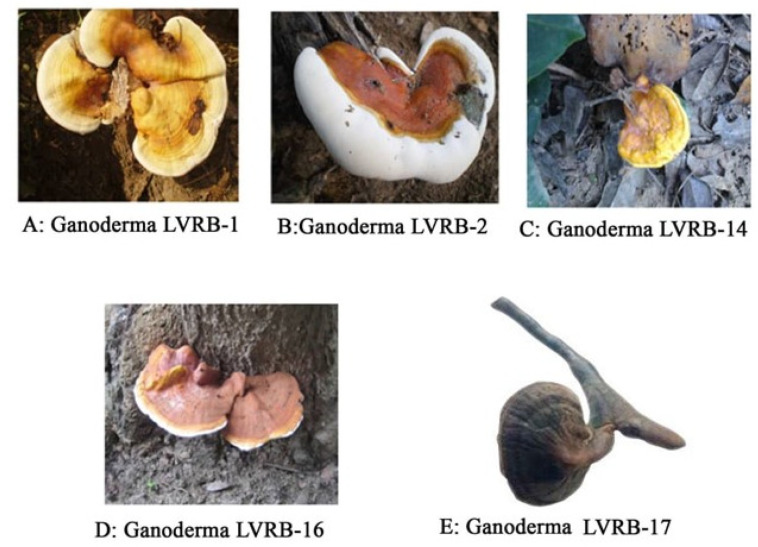
(**A**) Ganoderma isolate LVRB-1 growing on dead *Azadirachta indica* collected from a house in Agortigagorme. (**B**) Ganoderma isolate LVRB-2 growing on dead *Acacia* spp. collected from Degorme. (**C**) Ganoderma isolate LVRB-14 growing on dead *Baphia nitida* collected from a farm in Lukunu. (**D**) Ganoderma isolate LVRB-16 growing at the base of *Mangifera indica* collected from Lukunu. (**E**) Ganoderma isolate LVRB-17 growing on dead *Acacia* spp. collected from Azaglo Torkor.

Of the five collections, two isolates coded Ganoderma LVRB-2 (Figure 2B) and Ganoderma LVRB-17 (Figure 2E) were found growing on a dead *Acacia* spp. tree, whereas the isolate coded Ganoderma LVRB-16 (Figure 2D) was found growing on *Mangifera indica*. The collections designated Ganoderma LVRB-1 (Figure 2A) were found growing on *Azadirachta indica*, while the last collection, designated Ganoderma LVRB-14 (Figure 2C), was found growing on *Baphia nitida*.

The fruiting bodies of the collections from *M. indica* and *Acacia* spp. were reddish brown in color, while collections from *A. indica* and *B. nitida* were yellowish brown. The sample collection site, along the Lower Volta River Basin, was found to have extensive bodies of water (Volta River, Aklakpa, and Aklamador) and mostly undulating land covered by luxuriant vegetation (Figure 1).

### 3.2. Sequence Generation

Amplification and sequencing of the ITS2 region were carried out for all five Ganoderma isolates. The analysis, however, revealed that the ITS2 sequence was insufficient for resolving the phylogenetic position of Ganoderma LVRB-2 and Ganoderma LVRB-17. As a result, the complete ITS region was amplified and sequenced for these two isolates. Similarly, the nLSU region was amplified and sequenced for all five isolates. The ITS2, ITS, and nLSU sequences of the isolates are presented in Supplementary Table S1A, S1B and S1C, respectively.

### 3.3. DNA Sequence Comparisons

Blastn comparisons of the ITS2 sequence data revealed that Ganoderma LVRB-1, Ganoderma LVRB-14, and Ganoderma LVRB-16 showed the highest similarity (99.49%, 99.74%, and 99.74%), respectively, with *G. enigmaticum* (Supplementary Table S1A). As indicated in Supplementary Table S1A, Ganoderma LVRB-2 showed the highest similarity (100%) with *G. mbrekobenum*, while Ganoderma LVRB-17 also showed the highest similarity (99.48%) with *G. mbrekobenum*. Likewise, in the ITS Blastn search, Ganoderma LVRB-2 displayed the highest similarity (99.47%) with *G. mbrekobenum*, while Ganoderma LVRB-17 showed the highest similarity (99.4%) with *G. resinaceum* in the ITS Blastn search (Supplementary Table S1B). BLASTn search analysis of the nLSU sequence, however, revealed that Ganoderma LVRB-1, Ganoderma LVRB-14, and Ganoderma LVRB-16, like the ITS2, showed the highest similarity, 99.90%, 100.00%, and 99.71%, respectively, with *G. enigmaticum* (Supplementary Table S1C). As summarized in Supplementary Table S1C, Ganoderma LVRB-2 showed the highest similarity (100%) with *G. mbrekobenum*. Finally, Ganoderma LVRB-17 showed the highest similarity (100%) with *G. resinaceum* (Supplementary Table S1C).

### 3.4. ITS2 Phylogenetic Analysis

**Figure 3:**
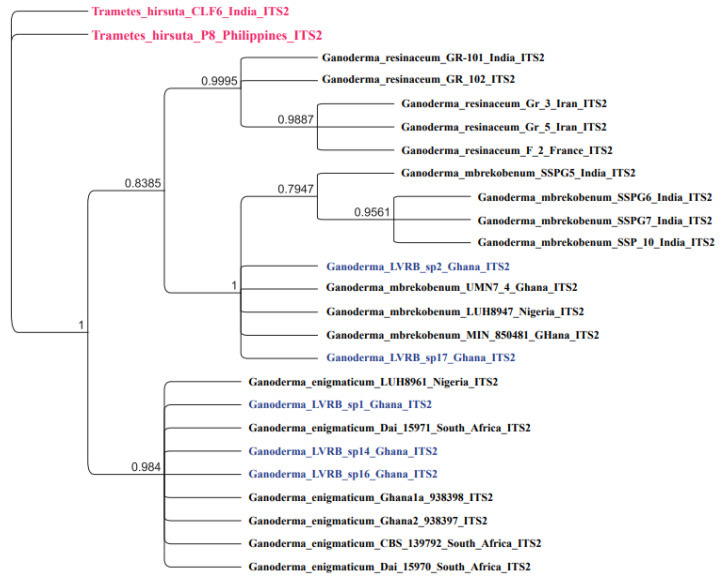
Bayesian phylogenetic tree showing the phylogenetic position of *Ganoderma* spp. collections from the Lower Volta River Basin of Ghana compared with available ITS2 rDNA sequence data of the *Ganoderma* genus in GenBank. *Trametes hirsuta* CLF6 and *Trametes hirsuta* P8 were used as outgroups. Numbers at the branch nodes represent BPP values.

Bayesian posterior probability (BPP) was conducted for all the sequenced *Ganoderma* spp. collections (n = 5) using MrBayes in Geneious Prime Version 2022.01, and the resulting tree is presented in Figure 3. As described in Figure 3, Ganoderma LVRB-1, Ganoderma LVRB-14, and Ganoderma LVRB-16 formed a well-supported clade (BPP = 0.984) with *Ganoderma enigmaticum*. As illustrated in Figure 3, Ganoderma LVRB-2 and Ganoderma LVRB-17 statistically clustered strongly with *G. mbrekobenum* (BPP = 1.00). The ITS2 Bayesian phylogenetic analysis revealed the five isolates are separated into two distinct clades, namely *G. enigmaticum* and *G. mbrekobenum* (Figure 3).

### 3.5. ITS Phylogenetic Analysis

**Figure 4:**
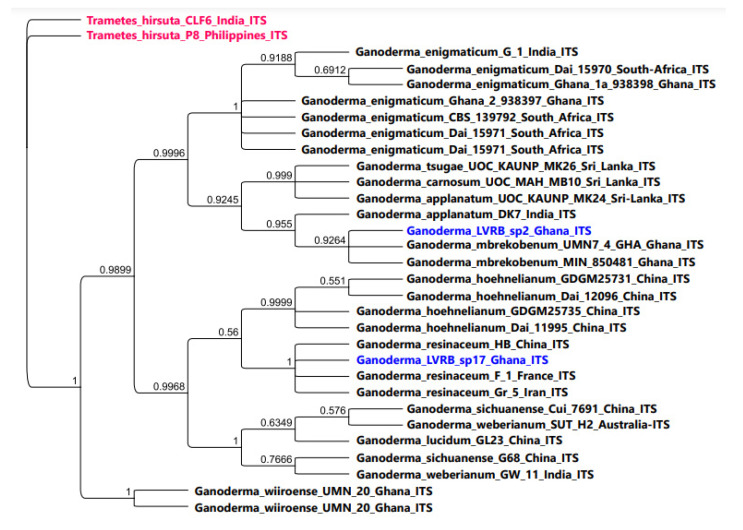
Bayesian phylogenetic tree showing the phylogenetic position of *Ganoderma* spp. collections from the Lower Volta River Basin of Ghana compared with available ITS rDNA sequence data of the genus *Ganoderma* in GenBank. *Trametes hirsuta* CLF6 and *Trametes hirsuta* P8 were used as outgroups. Numbers at the branch nodes represent BPP values.

The ITS Bayesian phylogenetic analysis of Ganoderma LVRB-2 and Ganoderma LVRB-17 is presented in Figure 4. As illustrated in Figure 4, Ganoderma LVRB-17 strongly clustered with *G. resinaceum* (BPP = 1.00).

As shown in Figure 4, Ganoderma LVRB-2 strongly clustered with two isolates of *G. mbrekonenum* from Ghana (BPP = 0.9264), thus providing further evidence that Ganoderma LVRB-2 belongs to *G. mbrekobenum*. The ITS Bayesian phylogenetic analysis demonstrated that Ganoderma LVRB-2 and Ganoderma LVRB-17 were separated into two clades, namely *G. mbrekobenum* and *G. resinaceum*, respectively (Figure 4).

### 3.6. nLSU Phylogenetic Analysis

**Figure 5:**
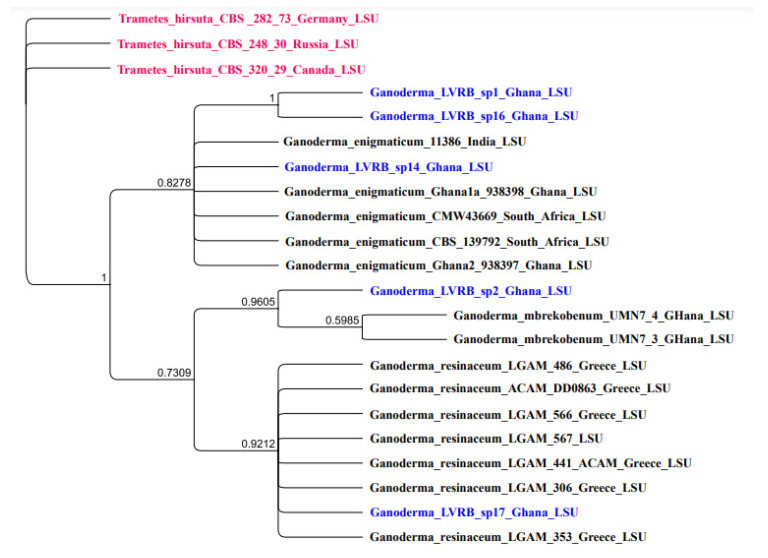
Bayesian posterior probability (BPP) tree showing the phylogenetic position of *Ganoderma* spp. collections from the Lower Volta River Basin of Ghana compared with available nLSU sequence data of the genus *Ganoderma* in GenBank. *Trametes hirsuta* CBS 282, *Trametes hirsuta* CBS 320, and *Trametes hirsuta* CBS 248 were used as outgroups. Numbers at the branch nodes represent BPP values.

The nLSU was sequenced for the five isolates, and the resultant phylogenetic tree is presented in Figure 5.

As illustrated in Figure 5, Ganoderma LVRB-2 strongly clustered with *G. mbrekobenum* (BPP = 0.9605), further confirming that Ganoderma LVRB-2 belongs to the species *G. mbrekobenum*. Ganoderma LVRB-17, on the other hand, clustered with *G. resinaceum* (BPP = 0.9212). The finding of nLSU phylogenetic analysis was consistent with the observation made in the ITS phylogenetic analysis, further suggesting that Ganoderma LVRB-17 belongs to the species *G. resinaceum*. The LSU phylogenetic tree generated from the Bayesian analysis of the LSU in this study also revealed that Ganoderma LVRB-1, Ganoderma LVRB-14, and Ganoderma LVRB-16 formed a clade with *G. enigmaticum* with high support (BPP = 0.8272), as observed in the ITS2 phylogenetic analysis. 

## 4. Discussion

Numerous studies have established that medicinal mushrooms have great therapeutic potential in human health as they possess many significant medicinal properties, including anti-diabetic, anticancer, anti-obesity, immunomodulatory, hypocholesteremia, hepatoprotective, and anti-aging [23]. Indeed, *Ganoderma* is one of the most extensively studied genera of medicinal mushrooms, and it has proven to be a panacea in alternative medicine and a significant reservoir of medically important bioactive compounds [24]. It is also worth mentioning that medicinal mushrooms represent an important source of functional enzymes with biocatalytic potential [25,26]. In the present study, we conducted a mycological survey and collected five novel *Ganoderma* isolates from different locations along the banks of the Lower Volta River Basin of Ghana, in West Africa. Previously, molecular phylogenetic analysis of the internal transcribed spacer 2 (ITS2) [19], complete internal transcribed spacer (ITS) [25], and nuclear large subunit (nLSU) [26,27,28] were used to identify *Ganoderma* spp. from different geographical regions of the world. Recently, *G. mbrekobenum*, a wood-rotting fungus originally isolated from Brong Ahafo and Greater Accra, was characterized using ITS and LSU genes and named after the Ghanaian Twi word ‘mbrekoben’, which translates to reddish brown mushroom [16]. In 2021, Parihar and coworkers identified *G. mbrekobenum* in India [29], while Ofodile et al., just one year ago, isolated *G. mbrekobenum* from Nigeria [30] based on ITS Blastn search and phylogenetic analysis. In the present study, we targeted three nuclear ribosomal sequences to characterize Ghanaian *Ganoderma* spp. using the Bayesian method instead of neighbor-joining (distance-based) and parsimony methods. The choice for the Bayesian method is because neighbor-joining (distance-based) and parsimony are known not to perform well with molecular sequences that are evolving at different rates [31]. Besides the above explanation, the Bayesian method is also known to provide a faster measure of clade support and differs from the maximum likelihood method in that it allows for complex models of nucleotide or amino acid evolution to be implemented [31].

The results of the ITS2, ITS, and nLSU Blastn search and molecular phylogenetic analysis revealed that the isolate designated Ganoderma LVRB-2 belongs to the species *G. mbrekobenum* originally isolated from the Brong Ahafo and Greater Accra regions of Ghana. Aside from Brong Ahafo and Greater Accra, *G. mbrekobenum* has not been reported in any other region of Ghana, making the current isolation from the Volta River Basin a very important observation. Since *G. mbrekobenum* from Ghana is not well studied, there is a need to comprehensively study Ghanaian *G. mbrekobenum* to help reveal its potential utilization in comparison with *Ganoderma* species from other geographical regions of the world. Recently, in search of medicinally active compounds in mushrooms of the genus *Ganoderma*, eleven undescribed lanostane triterpenoids were isolated from artificially cultivated fruiting bodies of *G. mbrekobenum* [32]. Interestingly, two of the undescribed lanostane triterpenoids exhibited moderate antimalarial activity. This finding suggests that Ganoderma LVRB-2, which formed a well-supported clade with *G. mbrekobenum* in this present study, may also have antimalarial activity. In another recent study, extracts of the *G. mbrekobenum* fruiting body collected from Tanzania effectively inhibited the proliferation of the human liver cancer cell line HepG2, the human breast cancer cell line MDA-MB-231, and the human brain cancer cell line U87 [33]. These findings suggest that *G. mbrekobenum* may prove to be a natural source of biological precursors for the development of antimalarial and anticancer drugs.

Similar to *G. mbrekobenum*, several medicinally active compounds could be present in the other *Ganoderma* species from Ghana. Coetzee et al., (2015) studied the *Ganoderma* species, including a new taxon associated with root rot of the iconic *Jacaranda mimosifolia* in Pretoria, South Africa. The phylogenetic trees, which were generated using DNA sequences obtained from the ITS and LSU regions of the ribosomal RNA operon, confirmed *G. enigmaticum* as a new species [34]. Recently, *G. enigmaticum* was reported in Nigeria using internal transcribed spacer sequences [32]. In the present study, ITS2 and nLSU Blastn search and molecular phylogenetic analysis demonstrated that Ganoderma LVRB-1, Ganoderma LVRB-14, and Ganoderma LVRB-16 belong to the *G. enigmaticum* described previously from the Pretoria Province of South Africa [34]. The second ever *G. enigmaticum* isolation reported in the literature was from the Upper West region of Ghana [16]. This current isolation of *G. enigmaticum* from the Lower Volta River Basin is very useful information and represents the second new record of *G. enigmaticum* in Ghana, although the third-ever isolation was made from Nigeria [30]. Recently, four lanostane triterpenoids were identified in the artificially cultivated mycelial biomass of *G. enigmaticum* from Ghana [35]. The identified lanostane compounds include ganoderic acid C6, ganoderenic acid D, ganoderenic acid A, and ganoderic acid G, together with ganoderenic acid K and ganoderenic acid AM1 as annotated compounds.

*G. resinaceum* is used in ethnomedicine for immune regulation and treating hyperglycemia and liver disease [36,37]. Recently, El-Fallal et al., (2015) confirmed the status of *Ganoderma* species collected in the Northeast Nile Delta, Egyptm as *G. resinaceum* by analyzing the ribosomal 5.8S rRNA gene and the flanking internal transcribed spacers (ITS) [38]. In the current study, ITS and nLSU molecular phylogenetic analyses revealed that the Ganoderma sample LVRB-17 clustered with the species *G. resinaceum*. The isolation of *G. resinaceum* in the present study represents the first molecular evidence of the occurrence of *G. resinaceum* in Ghana. The results of the ITS2 phylogenetic analysis, on the other hand, showed that Ganoderma LVRB-2 and Ganoderma LVRB-17 belong to the same species, suggesting ITS2 was not able to resolve the identity of Ganoderma LVRB-17 in the current study, contrary to the observations of some researchers [19,39] that suggest that ITS2 was suitable for identification of *Ganoderma* species. In the present study, ITS1 was not used because ITS1 and ITS2 have been reported to yield, to a large extent, similar results when used as DNA metabarcodes for fungi [40]. Since *Ganoderma* species from different geographic regions have different bioactive compounds and biological activities, the isolates identified in the present molecular phylogenetic analysis need to be properly characterized using metabolomics approaches and their bioactivities investigated to provide insight into their biopharmaceutical potentials, thereby unlocking their potential nutraceutical and biopharmaceutical applications.

## 5. Conclusions

The present study confirmed that the *Ganoderma* species collected from the Lower Volta River Basin in Ghana belong to three distinct species, namely *G. mbrekobenum*, *G. enigmaticum*, and *G. resinaceum*. The molecular phylogenetic analysis of *Ganoderma* species from the Lower Volta Basin in this present study was generally consistent with the earlier findings by the Food Research Institute of Ghana and the University of Minnesota, in the USA, in which the occurrence of *G. mbrekobenum* and *G. enigmaticum* was reported in Ghana. To the best of our knowledge, this is the first phylogenetic study that provides molecular evidence for the occurrence of *G. resinaceum* in Ghana. The current study has provided information that will be useful for future studies regarding the molecular evolution, biomedical implications, and phytopathogenic significance of *Ganoderma* species in Ghana.

## Data Availability

Data available upon request.

## Supplementary Materials

The following supporting information can be downloaded at https://www.mdpi.com/article/10.3390/jof10010006/s1, Table S1A: ITS2 sequence matching results of isolates Ganoderma LVRB-1, Ganoderma LVRB-2, Ganoderma LVRB-14, Ganoderma LVRB-16 and Ganoderma LVRB-17 from the Lower Volta River Basin of Ghana. Table S1B: ITS sequence matching results of isolates of isolate Ganoderma LVRB-2 and Ganoderma LVRB-17 from the Lower Volta River Basin of GhanaITS2 sequence. Table S1C: nLSU nucleotide sequence matching results of isolates Ganoderma LVRB-1, Ganoderma LVRB-2, Ganoderma LVRB-14, Ganoderma LVRB-16 and Ganoderma LVRB-17 from the Lower Volta River Basin of Ghana.

## References

[1] Benzie I.F.F., Wachtel-Galor S. (2011). Herbal Medicine: Biomolecular and Clinical Aspects.

[2] Ryvarden L., Iturriaga T. (2003). Studies in neotropical polypores 10. New polypores from Venezuela. Mycologia.

[3] Ainsworth G.C. (2008). Ainsworth & Bisby’s Dictionary of the Fungi.

[4] Wasser S.P., Coates P., Blackman M., Cragg G., Levine M., Moss J., White J. (2005). Encyclopedia of Dietary Supplements.

[5] Zhao R.-L., He Y.-M. (2018). Network pharmacology analysis of the anti-cancer pharmacological mechanisms of *Ganoderma lucidum* extract with experimental support using Hepa1-6-bearing C57 BL/6 mice. J. Ethnopharmacol..

[6] Batra P., Sharma A.K., Khajuria R. (2013). Probing Lingzhi or Reishi medicinal mushroom *Ganoderma lucidum* (Higher Basidiomycetes): A bitter mushroom with amazing health benefits. Int. J. Med. Mushrooms.

[7] Chen S., Xu J., Liu C., Zhu Y., Nelson D.R., Zhou S., Li C., Wang L., Guo X., Sun Y. (2012). Genome sequence of the model medicinal mushroom *Ganoderma lucidum*. Nat. Commun..

[8] Shiao M.-S. (2003). Natural products of the medicinal fungus *Ganoderma lucidum*: Occurrence, biological activities, and pharmacological functions. Chem. Rec..

[9] Fryssouli V., Zervakis G., Polemis E., Typas M.A. (2020). A global meta-analysis of ITS rDNA sequences from material belonging to the genus *Ganoderma* (Basidiomycota, Polyporales) including new data from selected taxa. MycoKeys.

[10] Richter C., Wittstein K., Kirk P.M., Stadler M. (2015). An assessment of the taxonomy and chemotaxonomy of *Ganoderma*. Fungal Divers..

[11] Welti S., Moreau P., Decock C., Danel C., Duhal N., Favel A., Courtecuisse R. (2015). Oxygenated lanostane-type triterpenes profiling in laccate *Ganoderma* chemotaxonomy. Mycol. Prog..

[12] Singh M., Bandana, Ahuja P.S. (1999). Isolation and PCR Amplification of Genomic DNA from Market Samples of Dry Tea. Plant Mol. Biol. Rep..

[13] Moncalvo J.-M. (2005). Molecular Systematics of *Ganoderma*: What Is Reishi?. Int. J. Med. Mushrooms.

[14] Obodai M., Mensah D.L.N., Fernandes A., Kortei N.K., Dzomeku M., Teegarden M., Schwartz S.J., Barros L., Prempeh J., Takli R.K. (2017). Chemical Characterization and Antioxidant Potential of Wild *Ganoderma* Species from Ghana. Molecules.

[15] Crous P., Wingfield M., Le Roux J., Richardson D., Strasberg D., Shivas R., Alvarado P., Edwards J., Moreno G., Sharma R. (2015). Fungal Planet description sheets: 371–399. Persoonia Mol. Phylogeny Evol. Fungi.

[16] Crous P.W., Wingfield M.J., Richardson D.M., Le Roux J.J., Strasberg D., Edwards J., Roets F., Hubka V., Taylor P.W., Heykoop M. (2016). Fungal Planet description sheets: 400–468. Persoonia.

[17] Aime M., Phillips-Mora W. (2005). The causal agents of witches’ broom and frosty pod rot of cacao (chocolate, *Theobroma cacao*) form a new lineage of *Marasmiaceae*. Mycologia.

[18] White T.J., Bruns T.D., Lee S., Taylor J., Innis M.A., Gelfand D.H. (1990). Amplification and direct sequencing of fungal ribosomal RNA genes for phylogenetics. PCR Protocols: A Guide to Methods and Applications.

[19] Liao B., Chen X., Han J., Dan Y., Wang L., Jiao W., Song J., Chen S. (2015). Identification of commercial *Ganoderma* (Lingzhi) species by ITS2 sequences. Chin. Med..

[20] Gardes M., Bruns T.D. (1993). ITS primers with enhanced specificity for basidiomycetes—Application to the identification of mycorrhizae and rusts. Mol. Ecol..

[21] Hopple J.S., Vilgalys R. (1994). Phylogenetic relationships among coprinoid txa and allies based on data from restriction site mapping of nuclear rDNA. Mycologia.

[22] Moncalvo J., Flood J., Bridge P.D., Holderness M. (2000). Systematics of *Ganoderma*. Ganoderma Diseases of Perennial Crops.

[23] Chaturvedi V.K., Agarwal S., Gupta K.K., Ramteke P.W., Singh M.P. (2018). *Medicinal mushroom*: Boon for therapeutic applications. 3 Biotech.

[24] Chen X., Veena R.K., Ramya H., Janardhanan K.K., George V. (2020). Gano oil: A novel antinociceptive agent extracted from *Ganoderma lucidum* inhibits paw oedema and relieves pain by hypnotic and analgesic actions of fatty acid amides. J. Ethnopharmacol..

[25] Tan H., Tang J., Li X., Liu T., Miao R., Huang Z., Wang Y., Gan B., Peng W. (2017). Biochemical Characterization of a Psychrophilic Phytase from an Artificially Cultivable Morel *Morchella importuna*. J. Microbiol. Biotechnol..

[26] Zhang Q., Miao R., Liu T., Huang Z., Peng W., Gan B., Zhang X., Tan H. (2019). Biochemical characterization of a key laccase-like multicopper oxidase of artificially cultivable *Morchella importuna* provides insights into plant-litter decomposition. 3 Biotech.

[27] Gunnels T., Creswell M., McFerrin J., Whittall J.B. (2020). The ITS region provides a reliable DNA barcode for identifying reishi/lingzhi (*Ganoderma*) from herbal supplements. PLoS ONE.

[28] Luangharn T., Karunarathna S.C., Mortimer P.E., Hyde K.D., Xu J. (2019). Additions to the knowledge of Ganoderma in Thailand: Ganoderma casuarinicola, a new record; and *Ganoderma thailandicum* sp. nov. MycoKeys.

[29] Parihar S.S., Sahu S., Gupta G., Prakash A. (2021). *Ganoderma mbrekobenum*: A pharmacologically Important Mushroom Naturally Growing in Raisen, India. Curr. Trends Biotechnol. Pharm..

[30] Ofodile L.N., Isikhuemhen O.S., Anike F.N., Adekunle A.A. (2022). The Domestication and Cultivation of *Ganoderma* (*Agaricomycetes*) Medicinal Mushroom Species from Nigeria. Int. J. Med. Mushrooms.

[31] Raja H.A., Miller A.N., Pearce C.J., Oberlies N.H. (2017). Fungal Identification Using Molecular Tools: A Primer for the Natural Products Research Community. J. Nat. Prod..

[32] Yangchum A., Fujii R., Choowong W., Rachtawee P., Pobkwamsuk M., Boonpratuang T., Mori S., Isaka M. (2022). Lanostane triterpenoids from cultivated fruiting bodies of basidiomycete *Ganoderma mbrekobenum*. Phytochemistry.

[33] Huiping H., Yuanchao L., Xiaowei L., Xiangmin L., Weipeng M., Yizhen X., Zhi Z., Qingping W. (2021). Artificial Cultivation Anti-tumor Activity of *Ganoderma mbrekobenum*. Sains Malays..

[34] Coetzee M.P.A., Marincowitz S., Muthelo V.G., Wingfield M.J. (2015). *Ganoderma* species, including new taxa associated with root rot of the iconic *Jacaranda mimosifolia* in Pretoria, South Africa. IMA Fungus.

[35] Adotey G., Alolga R.N., Quarcoo A., Gedel M.A., Anang A.K., Holliday J.C. (2021). Ultra Performance Liquid Chromatography-Quadrupole Time-of-Flight Mass Spectrometry (UPLC-Q-TOF-MS)-based metabolomic analysis of mycelial biomass of three *Ganoderma* isolates from the Lower Volta River Basin of Ghana. J. Pharm. Biomed. Anal..

[36] Peng X.-R., Liu J.-Q., Han Z.-H., Yuan X.-X., Luo H.-R., Qiu M.-H. (2013). Protective effects of triterpenoids from *Ganoderma resinaceum* on H_2_O_2_-induced toxicity in HepG2 cells. Food Chem..

[37] Oyetayo O.V. (2011). Medicinal uses of mushrooms in Nigeria: Towards full and sustainable exploitation. Afr. J. Tradit. Complement. Altern. Med..

[38] El-Fallal A. (2015). First record of two Ganoderma species from North East Nile DeltaEgypt. Mycosphere.

[39] Han J., Zhu Y., Chen X., Liao B., Yao H., Song J., Chen S., Meng F. (2013). The Short ITS2 Sequence Serves as an Efficient Taxonomic Sequence Tag in Comparison with the Full-Length ITS. BioMed Res. Int..

[40] Blaalid R., Kumar S., Nilsson R.H., Abarenkov K., Kirk P.M., Kauserud H. (2013). ITS1 versus ITS2 as DNA metabarcodes for fungi. Mol. Ecol. Resour..

