# Supplementary material for: Molecular Identification and Characterization of Five Ganoderma Species from the Lower Volta River Basin of Ghana Based on Nuclear Ribosomal DNA (nrDNA) Sequences
Source: J Fungi (Basel). 2023 Dec 21;10(1):6. doi: 10.3390/jof10010006 (PMC10817336; doi:10.3390/jof10010006)
Supplement: Supplementary file 1 [file jof-10-00006-s001.zip › jof-2738512-supplementary.pdf]

Supplementary Data

Table S1A: ITS2 sequence matching results of isolates Ganoderma LVRB-1, Ganoderma LVRB-2, Ganoderma LVRB-14, Ganoderma LVRB-16 and Ganoderma LVRB-17 from the Lower Volta River Basin of Ghana

Species ID	DNA sequence	Sequence similarity (%) showing species of top matching candidate
Ganoderma LVRB-1	GCATCGATGAAGAACGCAGCGAAATGCGATAAGTAATGTGAATTGCAGAATTCAGTGAATCATCGAATCTTTGAACGCACCT<br>TGCGCTCCTTGGTATTCCGAGGAGCATGCCTGTTTGAGTGTCATGAAATCTTCAACTTGCAACCTCTTTGCGGAGTTTGTAGGC<br>TTGGACTTGGAGGGCTTGTCGGCCTTTAACGGTCGGCTCCTCTTAAATGCATTAGCTTGATTCCTTGCRGATCGGCTGTCGGTG<br>TGATAAAATGTCTACGCCGTGACCGTGAAGCGTTTGGATGAGCTTCcAACCGTCTTGsTTCAAAGACAACTTTttATGACCTCTG<br>ACCTCAAATCAGGTAGGACTACCCGCTGAACTTAAGCATATCAATAAGCGGAGGA	*Ganoderma enigmaticum* voucher Ghana1a/938398<br><br>99.49%
Ganoderma LVRB-2	GCATCGATGAAGAACGCAGCGAAATGCGATAAGTAATGTGAATTGCAGAATTCAGTGAATCATCGAATCTTTGAACGCACCT<br>TGCGCTCCTTGGTATTCCGAGGAGCATGCCTGTTTGAGTGTCATGAAATCTTCAACCTGCAAGCTTTTAATCGGTTTGTAGGCT<br>TGGATTTGGAGGCTATTGTCGGCCTTTATCGGTCGGCTCCTCTTAAATGTATTAGCTTGGTTCCTTGCGGATCGGCTTGTCGGT<br>GTGATAATGTCTACGCCGCGACCGTGAAGCGTTTGGGCAAGCTTCTAACCGTCTCACTTTAGAGACAACCTTATGACCTCTGA<br>CCTCAAATCAGGTAGGACTACCCGCTGAACTTAAGCATATCAATAAGCGGAGGA	*Ganoderma mbrekobenum* voucher UMN7-4 GHA<br><br>100.00%
Ganoderma LVRB-14	GCATCGATGAAGAACGCAGCGAAATGCGATAAGTAATGTGAATTGCAGAATTCAGTGAATCATCGAATCTTTGAACGCACCT<br>TGCGCTCCTTGGTATTCCGAGGAGCATGCCTGTTTGAGTGTCATGAAATCTTCAACTTGCAACCTCTTTGCGGAGTTTGTAGGC<br>TTGGACTTGGAGGGCTTGTCGGCCWTTAACGGTCGGCTCCTCTTAAATGCATTAGCTTGATTCCTTGCGGATCGGCTGTCGGT<br>GTGATAAAATGTCTACGCCGTGACCGTGAAGCGTTTGGATGAGCTTCCAACCGTCTTGCTTCAAAGACAACTTTTTATGACCTC<br>TGACCTCAAATCAGGTAGGACTACCCGCTGAACTTAAGCATATCAATAAGCGGAGGA	*Ganoderma enigmaticum* voucher Ghana1a/938398<br><br>99.74%
Ganoderma LVRB-16	GCATCGATGAAGAACGCAGCGAAATGCGATAAGTAATGTGAATTGCAGAATTCAGTGAATCATCGAATCTTTGAACGCACCT<br>TGCGCTCCTTGGTATTCCGAGGAGCATGCCTGTTTGAGTGTCATGAAATCTTCAACTTGCAACCTCTTTGCGGAGTTTGTAGGC<br>TTGGACTTGGAGGGCTTGTCGGCCWTTAACGGTCGGCTCCTCTTAAATGCATTAGCTTGATTCCTTGCGGATCGGCTGTCGGT<br>GTGATAAAATGTCTACGCCGTGACCGTGAAGCGTTTGGATGAGCTTCcAACCGTCTTGCTTcAAaGACAaCTTTttATGACCTCTG<br>ACCTCAAATCAGGTAGGACTACCCGCTGAACTTAAGCATATCAATAAGCGGAGGA	*Ganoderma enigmaticum* voucher Ghana1a/938398<br><br>99.74%*
Ganoderma LVRB-17	GCATCGATGAAGAACGCAGCGAAATGCGATAAGTAATGTGAATTGCAGAATTCAGTGAATCATCGAATCTTTGAACGCACCT<br>TGCGCTCCTTGGTATTCCGAGGAGCATGCCTGTTTGAGTGTCATGAAATCTTCAACCTGCAAGCTTTTAATCGGTTTGTAGGCT<br>TGGATTTGGAGGCTATTGTCGGCCTTTATCGGTCGGCTCCTCTTAAATGTATTAGCTTGGTTCCTTGCGGATCGGCTTGTCGGT<br>GTGATAATGTCTACGCCGCGACCGTGAAGYGTTTGGGCAAGCTTCTAACCGTCTCACTTTAGAGCAACCTTATGACCTCTGAC<br>CTCAAATCAGGTAGGACTACCCGCTGAACTTAAGCATATCAATAAGCGGAGGA	*Ganoderma mbrekobenum* voucher UMN7-4<br><br>99.48%*

Table S1B: ITS sequence matching results of isolates of isolate Ganoderma LVRB-2 and Ganoderma LVRB-17 from the Lower Volta River Basin of GhanaITS2 sequence.

Species ID	DNA sequence	Sequence similarity (%) showing species of top matching candidate
Ganoderma LVRB-2	GTAAAAGTCGTAACAAGGTTTCCGTAGGTGAACCTGCGGAAGGATCATTATCGAGTTTTGACTGGGTTGTAGCTGGCCT<br>TACGAGGCATGTGCACGCCCTGCTCATCCGCTCTACACCTGTGCACTTACTGTGGGTTACAGACGGTGAAGCGGGCTTC<br>TTACGGGGAGCTTGTGAAGCGTGTCTGTGCCTGCGTTTACCACAAACTCTTTAAAGTATTAGAATGTGTATTGCGATGTA<br>ACGCATCTATATACAACTTTCAGCAACGGATCTTGGCTCTCGCATCGATGAAGAACGCAGCGAAATGCGATAAGTAATG<br>TGAATTGCAGAATTCAGTGAATCATCGAATCTTTGAACGCACCTTGCGCTCCTTGGTATTCCGAGGAGCAT	*Ganoderma mbrekobenum voucher UMN7-4 GHA*<br>99.49%
Ganoderma LVRB-17	GTAAAAGTCGTAACAAGGTTTCCGTAGGTGAACCTGCGGAAGGATCATTATCGAGTTTTGACTGGGTTGTAGCTGGCCT<br>TCCGAGGCATGTGCACACCCTGCTCATCCACTCTACACCTGTGCACTTACTGTGGGTTCCAGACGTTGTGAAGCGGGCT<br>CTTTACGGAGCTTGTAAAGCGGCGTGCCTGTGCCTGCGTTTATCACAAACTCTATAAAGTATTAGAATGTGTATTGCGAT<br>GTAACGCATCTATATACAACTTTCAGCAACGGCTCTTGGCTCTCGCATCGATGAAGAACGCAGCGAAATGCGATAAGTA<br>ATGTGAATTGCAGAATTCAGTGAATCATCGAATCTTTGAACGCACCTTGCGCTCCTTGGTATTCCGAGGAG	*Ganoderma resinaceum* isolate F-2<br>99.48%

Table S1C: nLSU nucleotide sequence matching results of isolates Ganoderma LVRB-1, Ganoderma LVRB-2, Ganoderma LVRB-14, Ganoderma LVRB-16 and Ganoderma LVRB-17 from the Lower Volta River Basin of Ghana

Species ID	DNA sequence	Sequence similarity (%) showing species of top matching candidate
Ganoderma LVRB-1	GAGCGGTCCAATCAAGCGACGGCTCGTTCTTACATATTTAAAGTTTGAGAATAGGTTAAGGTTGTTTCAACCCCAAGGCC<br>TCTAATCATTCGCTTTACCACATAAATCTGATAATGAGTTTCTGCTATCCTGAGGGAAACTTCGGCAGGAACCAGCTACTA<br>GATGGTTCGATTAGTCTTTCGCCCCTATACCCAAATTTGACGATCGATTTGCACGTCAGAATCGCTACGAGCCTCCACCAG<br>AGTTTCCTCTGGCTTCACCCTATTCAGGCATAGTTCACCATCTTTCGGGTCCCAACATACATGCTCTACCGCGGATCCGTC<br>AGAGAACGTCAGGTCCGGGCGTCGATGCCCCCCACGACAGGGGTCTCAACTTTCACTTTCATTACGCGCTCGGGTTTTCC<br>ACCCAAACACTCGCAGGTATGTTAGACTCCTTGGTCCGTGTTTCAAGACGGGTCGTTTAAAGCCATTATGCCAGCATCCTA<br>AGCGCGAAAGTGGGATAAACCCCTGCCTTACGGCGCGCTGCGTTCCTCGATCCCAACCGCCGTATGCGACCAGAGTCTAT<br>AACACACCATAAGGTGCCACATTACTCCAGCCCTTTTCCGACGGTCAAAATCGATGCTGACCCGTCAATCCGGAAAGTGC<br>ACCAAGCAAAAAAGCAAGGCTGAGTTCCGGATGACGCGACTGACTTCAAGCGTTTCCCTTTCAGCAATTTCACGTACTGT<br>TTAACTCTCTTTCCAAAGTGCTTTTCATCTTTCCCTCACGGTACTTGTTCGCTATCGGTCTCTCGCCAATATTTAGCTTTAG<br>ATGGAATTCACCACCCATTTTGAGCTGCATTCCCAAACAACTCGACTCTTTGAGAGCGCATCACAAAGCACTGGTAGTCC<br>GTGTCAAAGACGGGATTCTCACCCTCTATGACGCTCTGTTCCAAGAGACTTATACACGGTCCAGCGCGGAAAGCACTTCT<br>CCAGACTACAACTCGGACGGCCAAAGACCGCCAGATTTTAAATTTGAGCTTTTCCCGCTTCACTCGCAGTTACTAGGGGA<br>ATC	*Ganoderma enigmaticum* CBS 139792<br><br>99.90%
Ganoderma LVRB-2	TAAGCATATCAATAAGCGGAGGAAAAGAAACTAACAAGGATTCCCCTAGTAACTGCGAGTGAAGCGGGAAAAGCTCAA<br>ATTTAAAATCTGGCGGTCTTTGGCCGTCCGAGTTGTAGTCTGGAGAAGTGCTTTCCGCGCTGGACCGTGTATAAGTCTCTT<br>GGAACAGAGCGTCATAGAGGGTGAGAATCCCGTCTTTGACACGGACTACCAGTGCTTTGTGATGCGCTCTCAAAGAGTCG<br>AGTTGTTTGGGAATGCAGCTCAAAATGGGTGGTGAATTCCATCTAAAGCTAAATATTGGCGAGAGACCGATAGCGAACA<br>AGTACCGTGAGGGAAAGATGAAAAGCACTTTGGAAAGAGAGTTAAACAGTACGTGAAATTGCTGAAAGGGAAACGCTT<br>GAAGTCAGTCGCGTTGTCCGGAACTCAGCCTTGCTTTTGCTTGGTGCACTTTCCGGATGACGGGTCAGCATCGATTTTGAC<br>CGTCGGAAAAGGGCTAGAGTAATGTGGCACCTTCGGGTGTGTTATAGACTCTGGTCGCATACGGCGGTTGGGATCGAGG<br>AACGCAGCGCGCCGTAAGGCAGGGGTTCTCCCACTTTCGCGCTTAGGATGCTGGCATAATGGCTTTAAACGACCCGTCT	*Ganoderma mbrekobenum* voucher UMN7-4 GHA<br><br>100.0%
Ganoderma LVRB-14	GATTCCCCTAGTAACTGCGAGTGAAGCGGGAAAAGCTCAAATTTAAAATCTGGCGGTCTTTGGCCGTCCGAGTTGTAGTC<br>TGGAGAAGTGCTTTCCGCGCTGGACCGTGTATAAGTCTCTTGGAACAGAGCGTCATAGAGGGTGAGAATCCCGTCTTTGA<br>CACGGACTACCAGTGCTTTGTGATGCGCTCTCAAAGAGTCGAGTTGTTTGGGAATGCAGCTCAAAATGGGTGGTGAATTC<br>CATCTAAAGCTAAATATTGGCGAGAGACCGATAGCGAACAAGTACCGTGAGGGAAAGATGAAAAGCACTTTGGAAAGA<br>GAGTTAAACAGTACGTGAAATTGCTGAAAGGGAAACGCTTGAAGTCAGTCGCGTCGTCCGGAACTCAGCCTTGCTTTTTT<br>GCTTGGTGCACTTTCCGGATTGACGGGTCAGCATCGATTTTGACCGTCGGAAAAGGGCTGGAGTAATGTGGCACCTTATG<br>GTGTGTTATAGACTCTGGTCGCATACGGCGGTTGGGATCGAGGAACGCAGCGCGCCGTAAGGCAGGGGTTTATCCCACTT<br>TCGCGCTTAGGATGCTGGCATAATGGCTTTAAACGACCCGTCTTGAAACACGGACCAAGGAGTCTAACATACCTGCGAGT<br>GTTTGGGTGGAAAACCCGAGCGCGTAATGAAAGTGAAAGTTGAGACCCCTGTCGTGGGGGGCATCGACGCCCGGACCTG<br>ACGTTCTCTGACGGATCCGCGGTAGAGCATGTATGTTGGGA	*Ganoderma enigmaticum* voucher Ghana1a/938398<br><br>100.00%

Ganoderma LVRB-16	GATTCCCCTAGTAACTGCGAGTGAAGCGGGAAAAGCTCAAATTTAAAATCTGGCGGTCTTTGGCCGTCCGAGTTGTAGTC<br>TGGAGAAGTGCTTTCCGCGCTGGACCGTGTATAAGTCTCTTGGAACAGAGCGTCATAGAGGGTGAGAATCCCGTCTTTGA<br>CACGGACTACCAGTGCTTTGTGATGCGCTCTCAAAGAGTCGAGTTGTTTGGGAATGCAGCTCAAAATGGGTGGTGAATTC<br>CATCTAAAGCTAAATATTGGCGAGAGACCGATAGCGAACAAGTACCGTGAGGGAAAGATGAAAAGCACTTTGGAAAGA<br>GAGTTAAACAGTACGTGAAATTGCTGAAAGGGAAACGCTTGAAGTCAGTCGCGTCGTCCGGAACTCAGCCTTGCTTTTTT<br>GCTTGGTGCACTTTCCGGATTGACGGGTCAGCATCGATTTTGACCGTCGGAAAAGGGCTGGAGTAATGTGGCACCTTATG<br>GTGTGTTATAGACTCTGGTCGCATACGGCGGTTGGGATCGAGGAACGCAGCGCGCCGTAAGGCAGGGGTTTATCCCACTT<br>TCGCGCTTAGGATGCTGGCATAATGGCTTTAAACGACCCGTCTTGAAACACGGACCAAGGAGTCTAACATACCTGCGAGT<br>GTTTGGGTGGAAAACCCGAGCGCGTAATGAAAGTGAAAGTTGAGACCCCTGTCGTGGGGGGCATCGACGCCCGGACCTG<br>ACGTTCTCTGACGGATCCGCGGTAGAGCATGTATGTTGGGA	*Ganoderma enigmaticum* CBS 139792	99.71%
Ganoderma LVRB-17	GATTCCCCTAGTAACTGCGAGTGAAGCGGGAAAAGCTCAAATTTAAAATCTGGCGGTCTTTGGCCGTCCGAGTTGTAGTC<br>TGGAGAAGTGCTTTCCGCGCTGGACCGTGTATAAGTCTCTTGGAACAGAGCGTCATAGAGGGTGAGAATCCCGTCTTTGA<br>CACGGACTACCAGTGCTTTGTGATGCGCTCTCAAAGAGTCGAGTTGTTTGGGAATGCAGCTCAAAATGGGTGGTGAATTC<br>CATCTAAAGCTAAATATTGGCGAGAGACCGATAGCGAACAAGTACCGTGAGGGAAAGATGAAAAGCACTTTGGAAAGA<br>GAGTTAAACAGTACGTGAAATTGCTGAAAGGGAAACGCTTGAAGTCAGTCGCGTCGTCCGGAACTCAGCCTTGCTTTTTT<br>GCTTGGTGCACTTTCCGGATTGACGGGTCAGCATCGATTTTGACCGTCGGAAAAGGGCTGGAGTAATGTGGCACCTTATG<br>GTGTGTTATAGACTCTGGTCGCATACGGCGGTTGGGATCGAGGAACGCAGCGCGCCGTAAGGCAGGGGTTTATCCCACTT<br>TCGCGCTTAGGATGCTGGCATAATGGCTTTAAACGACCCGTCTTGAAACACGGACCAAGGAGTCTAACATACCTGCGAGT<br>GTTTGGGTGGAAAACCCGAGCGCGTAATGAAAGTGAAAGTTGAGACCCCTGTCGTGGGGGGCATCGACGCCCGGACCTG<br>ACGTTCTCTGACGGATCCGCGGTAGAGCATGTATGTTGGGA	*Ganoderma resinaceum* voucher LGAM 566	100.00%
